# Circulating tumor DNA for MRD detection in colorectal cancer: recent advances and clinical implications

**DOI:** 10.1186/s40364-025-00796-w

**Published:** 2025-06-23

**Authors:** Dening Ma, Xinyi Gao, Li Wang, Huan Yin, Longhai Feng, Yuping Zhu

**Affiliations:** 1 Department of Colorectal Surgery, Zhejiang Cancer Hospital, Hangzhou, 310022 China; 2 Department of Radiology, Zhejiang Cancer Hospital, Hangzhou, 310022 Zhejiang China; 3 Geneplus-Beijing Institute, Beijing, 102206 China

**Keywords:** Circulating tumor DNA, Molecular residual disease, Biomarker, Colorectal cancer

## Abstract

Circulating tumor DNA (ctDNA)-based molecular residual disease (MRD) provides a powerful approach to predict recurrence in colorectal cancer (CRC) and potentially improve survival outcomes for individuals diagnosed with CRC. Currently, there are two primary technical approaches for the detection of MRD using ctDNA: the tumor-informed assays and the tumor-agnostic assays. Multiple studies have demonstrated the role of MRD detection in CRC patients after radical therapy, including early relapse monitoring, molecular profiling, and treatment response prediction. Numerous interventional clinical trials based on ctDNA are underway to explore the value of MRD in optimizing adjuvant treatment decisions for patients with CRC. Once validated, ctDNA-MRD has the potential to impact current clinical treatment decisions. In this review, we summarize current techniques for detecting MRD based on ctDNA and review the data that have been collected to date on MRD detection in CRC patients who received curative-intent therapy. We also discuss prospective research of ctDNA MRD detection in this patient population and provide guidelines for the current and future use of MRD in clinical practice.

## Background

Colorectal cancer (CRC) is the third most common and second most lethal cancer-related malignancy in the world [1]. In recent years, there has been a rapid increase in incidence in China [2]. Approximately 517,100 people are diagnosed with CRC each year in China [3]. Patients with early-stage CRC can be cured by surgery, but 30% of them experience relapse [4]. Molecular residual disease (MRD) refers to trace amounts of tumor DNA fragments that remain in the blood or other body fluids after surgical resection or treatment and are considered the main cause of CRC local recurrence and distal metastasis [5, 6]. Currently, the diagnosis of CRC relies on histopathology and imaging, but these methods have limitations and cannot accurately reflect the presence of MRD in the late-stage or recurrence and metastasis of colorectal cancer. Therefore, a biomarker that can accurately identify patients with MRD is of critical importance.

In the past few years, with the development of molecular biology techniques, liquid biopsy has become an emerging method to detect MRD [7]. At present, circulating tumor DNA (ctDNA) in the blood serves as a reliable biomarker for assessing the presence of MRD. The most commonly used method for ctDNA-MRD detection is tumor-specific mutations [8, 9]. In addition, the ctDNA assays also include blood-based tumor mutation burden, methylation profiles, and genome-wide copy number alterations for quantitative and qualitative analyses that provide valuable information for prognostic assessment [10–13]. Moreover, ctDNA can capture information from both primary tumors and metastatic sites and has the advantages of being non-invasive, dynamic, real-time, and comprehensive [8, 14]. This review summarizes the current research status of ctDNA and MRD in colorectal cancer and discusses the possible barriers and challenges.

### ctDNA MRD detection technologies

The level of ctDNA in the blood of early-stage patients is much lower than that in advanced-stage patients. A median ctDNA fraction of 10% is found in advanced-stage patients, while it is less than 0.1% in early-stage patients [15]. Especially, most early-stage patients experience a drop in ctDNA due to surgical resection or chemotherapy [16], which requires a highly sensitive assay for analyzing ctDNA-MRD. The original ctDNA assay was based on polymerase chain reaction (PCR) technology, which amplifies specific DNA sequences of cancer cells [17]. Droplet Digital PCR (ddPCR) utilizes the water-oil emulsion droplet system to conduct digital PCR, which is more sensitive than traditional PCR [18, 19]. However, the limitations of PCR-based MRD assays are that they require prior knowledge of the target sequence and can only detect one or a few mutations [17]. In contrast, next-generation sequencing (NGS) can sequence more DNA fragments at once, providing comprehensive information [20]. The emergence of novel technologies in MRD detection has been constant in recent times. To date, there are two methods widely used in detecting MRD, including tumor-informed assays and Tumor-agnostic assays (Table 1).

**Table 1:** CtDNA approaches for MRD assessment

Characteristic	Tumor-Informed					Tumor-agnostic			
Representative products	ddPCR [91]	Signatera [21]	Safe-SeqS [45]	FoundationOne^®^ Tracker [73]	MRDetect [11]	CAPP-Seq [62]	Guardant Reveal™ [34]	IDEA-France (WIF1 + NPY) [48]	
Target Alterations in Plasma DNA	1–3 variants with the highest VAF in tumor	16 somatic variants	One tumor-specific mutation in each patient	16 clonal somatic variants	Tumor-specific variants detected from WGS	a customized panel of 1021 genes	Somatic and epigenetic aberrations	*WIF1* and *NPY* gene hypermethylation	
Timing of post-op liquid biopsy	4–12 weeks	30 days	4–10 weeks	Median 26.5 day	Median 43 day	Within 1 month	11–148 days	Median 42 day	
Sensitivity	100%	88%	48%	69%	84%	54.5%	91%	89.9%	
Specificity	83.3%	98%	98%	100%	99%	100%	100%	33.6%	

Abbreviations: ddPCR, droplet digital PCR; Safe-SeqS, Safe-sequencing system, CAPP-Seq, Cancer Personalized Profiling by deep Sequencing; Post-op, post-operative

### Tumor-informed assays

Tumor-informed assays require genomic sequencing of tumor tissues to identify individual patients’ mutations. Subsequently, the mutations in the patient’s blood are detected and compared with those in tumor tissue samples to accurately detect MRD. Typical products for tumor-informed assays include Signatera, Safe SeqS, FoundationOne^®^Tracker and, MRDetect, among others. Signatera first performs whole exome sequencing (WES) on tumor tissue, from which 16 somatic variants are selected for personalized panel customization [8]. The presence of at least 2 variants in plasma samples is considered MRD positive, while those with fewer than 2 variants are considered negative [21]. The detection limit of Signatera is 0.01% variant allele frequency (VAF). The Safe-SeqS method utilizes a unique molecular identifier (UMI) to detect mutations in DNA templates [22]. A UMI is added to each original DNA fragment before amplification, and the amplification products are then sequenced. This effectively distinguishes between rare mutations and technical errors [22, 23]. Other commercial products used for detecting MRD include FoundationOne^®^Tracker, which utilizes a comprehensive genomic profiling (CGP)-informed approach, as well as MRDetect, which employs a whole genome sequencing (WGS) tumor informed approach [24, 25]. All these products have been validated in clinical cohorts [11, 26–28].

Tumor-informed assays detect specific mutations in the patient’s tumor tissue, reducing false-positive results and enhancing sensitivity and accuracy [29]. However, it also has some limitations. Due to the spatiotemporal heterogeneity of tumors, the samples used in tissue testing can only capture tumor information from a limited region. The genomic profile of the primary tumor may also change with disease progression and clinical treatment, which could lead to false negatives [30–32]. Additionally, Tumor-informed assays rely on tissue, have longer detection cycles, and incur higher costs.

### Tumor-agnostic assays

Most ctDNA-based MRD assays require a tissue biopsy to identify tumor-specific mutations, which may not be feasible or available for all patients [33, 34]. Tumor-agnostic assays can be based solely on blood plasma, eliminating the need to obtain tumor tissue. Typical products for Tumor-agnostic assays include Cancer Personalized Profiling by deep sequencing (CAPP-seq), Guardant Reveal^®^™. CAPP-seq has unique barcodes that can improve sensitivity and specificity, with detection limits as low as 0.01% [35]. CAPP-Seq has been applied for various clinical purposes, such as early detection, monitoring treatment response and resistance, and identifying actionable mutations [36–38]. Guardant Reveal^®^™ is based on the Shield™ technology platform, which utilizes a UMI approach to detect rare variants in ctDNA. The test analyzes over 1000 genomic regions and more than 2000 methylation sites, as well as protein biomarkers such as carcinoembryonic antigen (CEA), to achieve high sensitivity and specificity for detecting MRD and predicting recurrence in CRC patients without prior knowledge of tumor genomics [34].

Tumor-agnostic assays typically utilize fixed panels that cover more sites than Tumor-informed assays and can detect evolving variants in the blood [39]. These assays have a shorter detection time, lower cost, and better operability. However, the tumor-agnostic assays cannot capture enough patient-specific variations and have a lower sensitivity and a higher risk of false positives compared to the tumor-informed panel [32].

### Limitations of ctdna assays

At present, there are still many challenges in the detection of MRD in CRC clinical practice. Firstly, more than 50% of cell-free DNA (cfDNA) mutations are associated with clonal hematopoiesis (CH), rather than originating from tumor cells [40]. This suggests that genomic mutations from hematopoietic cells may also be detected in plasma, leading to false positives in ctDNA detection. Secondly, the existing MRD detection technology possesses distinct characteristics, and a standardized detection scheme is currently lacking. One of the most significant trends in the future is the improvement of technology’s reliability and repeatability.

ctDNA MRD for Resectable Early-Stage Colorectal Cancer.

For individuals diagnosed with early-stage colorectal cancer, MRD detection is primarily used to monitor recurrence following radical surgery and guide postoperative adjuvant treatment. Surgery is the standard treatment for patients diagnosed with resectable colorectal cancer. While the majority of patients with stage I and II CRC achieve favorable outcomes through surgery alone, approximately 4-40% of patients may still experience recurrence and metastasis following surgery [41–43]. Multiple studies have provided evidence for monitoring recurrence of ctDNA-MRD, emphasizing its potential for clinical applications (Table 2). Tie et al. [44] reported a landmark study, which is one of the largest early studies in this field. This study enrolled 230 patients diagnosed with stage II colon cancer and demonstrated the clinical efficiency of ctDNA MRD using a tumor-informed safety-SeqS assay. The study revealed that among 178 patients who did not receive adjuvant chemotherapy (ACT), those with postoperative MRD positivity had a recurrence rate of 79% during the follow-up period, while only 9.8% of MRD-negative patients experienced recurrence. Moreover, MRD positivity after chemotherapy was also associated with reduced RFS in patients who received chemotherapy (HR, 11; 95% CI, 1.8–6.8; *P* = 0.001). Tie et al. demonstrated that detectable ctDNA after surgery had a significantly lower recurrence-free survival (RFS) compared to those with undetectable ctDNA (HR, 3.8; 95% CI, 2.4–21.0; *P* < 0.001) in stage III CRC. In addition, patients who exhibited detectable ctDNA levels after chemotherapy (*n* = 15) demonstrated a 3-year RFS rate of only 30%, whereas those with undetectable ctDNA levels (*n* = 73) showed a significantly higher rate of 77% (HR, 6.8; 95% CI, 11.0-157.0; *P* < 0.001) [45]. A prospective multicenter study was conducted on 130 stage I-III colon cancer patients to detect MRD using the Signatera assay [21]. This study indicated that patients who had a positive MRD test result 30 days after surgery had a 7.2 times higher risk of relapse compared to those with negative results. After adjuvant chemotherapy, MRD-positive patients have a 17.5-fold increased risk of recurrence compared to those who test negative. Similarly, ctDNA-positive patients during follow-up surveillance had a 43.5 times higher risk of recurrence compared to ctDNA-negative patients. Moreover, ctDNA demonstrated a median lead time of 8.7 months (range, 0.8–16.5 months) compared to imaging in predicting recurrence.

**Table 2:** CtDNA MRD studies in early-stage CRC

Reference	Sample Size	Cancer Stage	Analysis Method	Neoadjuvant Chemotherapy	Adjuvant Chemotherapy	% of Patients ctDNA Positive	Main Findings
Chen et al., 2021 [6]	240	II-III	Geneseeq Prime™ 425-gene panel	0%	72.5%	Post-op: 8.3% Post-Treatment: 8.7% Surveillance: 20%	• MRD-positive was associated with high recurrence risk at day 3–7 post-operation (HR, 10.98; 95% CI, 5.31–22.72; *P* < 0.001), at the first sampling point after ACT (HR, 12.76; 95% CI, 5.39–30.19; *P* < 0.001), and during surveillance after definitive therapy (HR, 32.02; 95% CI, 10.79–95.08; *P* < 0.001). • Serial ctDNA analyses identified recurrence with an accuracy of 92.0% and could detect disease recurrence ahead of radiological imaging with a mean lead time of 5.01 months.
Henriksen et al., 2022 [91]	168	III	Signatera	0%	13.1%	Post-op: 14% Post-Treatment: 10.7% Surveillance: 15.0%	Detection of MRD was a strong recurrence predictor postoperatively (HR, 7.0; 95% CI, 3.7–13.5; *P* < 0.001) and directly after ACT (HR, 50.76; 95% CI, 15.4–167; *P* < 0.001).
Jin et al., 2021 [92]	73	I-IV	mqMSP	83.6%	84.9%	Pre-op:89.0% Post-op: 28.8% Surveillance: 47.4%	Postoperative detection of MRD was associated with poorer RFS (HR, 4.20; *P* = 0.0005).
Kotaka et al., 2023 [49] (GALAXY)	1039	II-IV	Signatera	0%	42.2%	Post-op 4week: 18% Post-op 12week: 13.8%	Patients with high-risk stage II or III CRC and positive MRD 4-week post-op derived benefit from ACT (HR, 6.59; *P* < 0.0001).
Parikh et al., 2019 [93]	72	0-III	Guardant Health NGS	Not reported	41.7%	Post-op: 19% Post-Treatment: 22.2% Surveillance: not reported	Patients with positive MRD after standard therapy completion had a recurrence PPV of 93%, and NPV of 80% (HR, 11.29; *P* < 0.0001).
Parikh et al., 2021 [33]	103	I-IV	Guardant Reveal assay	45.2%	54.8%	Post-op: not reported Post-Treatment: 24%	• Patients with detectable MRD after definitive therapy had a recurrence (PPV = 100%; HR, 11.28; *P* < 0.0001). • Integrating epigenomic signatures increased sensitivity by 25-36% versus genomic alterations alone.
Reinert et al., 2019 [21]	130	I-III	Signatera	Not reported	62%	Post-op: 10.6% Post-Treatment: 12% Surveillance: 20%	*•* Patients with longitudinal positive MRD post-therapy were at 40 times risk of recurrence vs. undetectable ctDNA (HR, 43.5; 95% CI, 9.8 to 193.5; *P* = 0.001). • Serial ctDNA analyses revealed disease recurrence up to 16.5 months ahead of standard-of-care radiologic imaging (mean, 8.7 months; range, 0.8–16.5 months).
Schøler et al., 2017 [94]	45	I-IV	ddPCR	Not reported	36.8%	Post-op: 28.6% (stages I–III) Post-Treatment: not reported Surveillance: not reported	*•* All 6 MRD-positive patients (I-III) relapsed within 3 months of surgery (HR, 37.7; 95% CI; 4.2–335.5; *P* < 0.001), with a median lead time of 9.4 months. *•* Patients with liver metastasis with positive MRD after curative therapy were at high risk of relapse (HR, 4.9; *P* = 0.007).
Suzuki et al., 2020 [95]	44	II-III	ddPCR	Not reported	36.4%	Post-op: not reported Post-Treatment: not reported Surveillance: not reported	Patients with positive MRD post-operation showed significantly shorter RFS compared to the patients with MRD negative (HR, 14.9; 95% CI, 0.7-313.5; *P* < 0.0001).
Taieb et al., 2021 [47]	1017	III	ddPCR	Not reported	100%	Post-op: 13.8% Post-Treatment: not reported Surveillance: not reported	*•* The 3-year DFS rate was 66.39% for MRD-positive patients and 76.71% for MRD-negative patients (*P* = 0.015). *•* MRD was confirmed as an independent prognostic marker for DFS (HR, 1.55, 95% CI 1.13–2.12, *P* = 0.006) and OS (HR, 1.65, 95% CI 1.12–2.43, *P* = 0.011).
Tarazona et al., 2019 [96]	150	I-III	ddPCR	Not reported	37.2%	Post-op: 20.3% Post-chemotherapy: 28% Surveillance: Not reported	*•* MRD detection after surgery and during follow-up was associated with poorer DFS (HR, 17.56; log-rank *P* = 0.0014 and HR, 11.33; log-rank *P* = 0.0001, respectively) with a median lead time of 11.5 months. *•* ctDNA after therapy was associated with early relapse (HR, 10.02; log-rank *P* < 0.0001).
Tie et al., 2016 [44]	230	II	Safe-SeqS	0%	23%	Post-op: 7.9% Post-Treatment: 11% Surveillance: 11.7%	• MRD was detected postoperatively in 14 of 178 (7.9%) patients, 11 (79%) of whom had recurred at a median follow-up of 27 months; recurrence occurred in 16 (9.8%) of 164 patients with MRD-negative (HR, 18; 95% CI, 7.9 to 40; *P* < 0.001). • ctDNA after completion of chemotherapy was associated with an inferior RFS (HR, 11; 95% CI, 1.8 to 68; *P* = 0.001).
Tie et al., 2019 [45]	96	III	Safe-SeqS	0%	100%	Post-op: 21% Post-Treatment: 17% Surveillance: not reported	*•* The 3-year RFI was 30% for detectable ctDNA patients after chemotherapy and 77% for undetectable ctDNA patients (HR, 6.8; 95% CI, 11.0-157.0; *P* < 0.001). *•* Postsurgical ctDNA was independently associated with RFI after adjusting for known clinicopathologic risk factors (HR, 7.5; 95% CI, 3.5–16.1; *P* < 0.001).
Tie et al., 2022 [51] (DYNAMIC)	455	II	Safe-SeqS	0%	ctDNA-guided group:15% standard-management group:28%	Post-op: 15% Post-Treatment: not reported Surveillance: not reported	ctDNA-guided management was found to be noninferior to standard management, with a lower percentage of patients receiving adjuvant chemotherapy than the standard-management group.
Wang et al., 2019 [97]	58	I-III	Safe-SeqS	0%	31%	Post-op: 22.4% Post-Treatment: 11% Surveillance: not reported	• Patients with positive MRD after surgery had 77% relapse vs. 0% of patients with negative MRD. • ctDNA-positive was 3 months earlier than the radiological and clinical evidence indicating relapse.

Abbreviations: CRC, colorectal cancer; ctDNA, circulating tumor DNA; MRD, molecular residual disease; Safe-SeqS, Safe-sequencing system; NGS, Next-generation sequencing; ddPCR, droplet digital PCR; mqMSP, methylation-specific quantitative PCR assay; HR, hazard ratio; CI, confidence interval; DFS, disease-free survival; RFS, recurrence-free survival; OS, overall survival; RFI, recurrence-free interval; Pre-op, pre-operative; Post-op, post-operative; ACT, adjuvant chemotherapy; PPV, positive predictive value; NPV, negative predictive value

The use of adjuvant chemotherapy can improve patients’ survival rates. However, study shows that even without adjuvant therapy, more than 50% of patients may still be cured [46]. MRD may influence decisions regarding the use of adjuvant chemotherapy. A tumor agnostic technique was validated on a cohort of stage III colorectal cancer patients from IDEA-France trial [47]. Patients who were MRD-negative had a better disease-free survival (DFS) (HR, 1.55; 95% CI, 1.13–2.12; *P* = 0.006) and overall survival (OS) (HR,1.65; 95% CI, 1.12–2.43; *P* = 0.011) than those who were MRD-positive. MRD-positive patients who received 3 months of adjuvant chemotherapy had a worse prognosis compared to those who received 6 months, highlighting the significance of MRD as a prognostic marker and its potential impact on decisions regarding adjuvant chemotherapy.

GALAXY is a prospective, large-scale observational study designed to monitor the tumor-informed, personalized ctDNA assay detected ctDNA in patients with resectable CRC (*n* = 1,039) [48]. A personalized and tumor-informed assay (Signatera, Natera, Inc.) was used for the detection and quantification of ctDNA in plasma samples. In this cohort, ctDNA-positive after surgery was associated with a higher risk of recurrence (HR, 10.0; *P* < 0.0001) and was the most significant prognostic factor for recurrence risk in patients with stage II or III CRC. Furthermore, patients who are ctDNA-positive postoperatively can benefit from adjuvant chemotherapy (HR, 6.59; *P* < 0.0001), no statistically significant benefit from ACT was observed in patients with negative ctDNA [49]. This suggests that postoperative MRD can be used as a biomarker to guide adjuvant therapy. In 2024, updated data from the GALAXY study [50] showed that ctDNA-positive patients had significantly shorter DFS compared to ctDNA-negative patients, with 24-month DFS rates of 28.9% and 85.9%, respectively.

Australian DYNAMIC study is the first MRD-directed prospective intervention study [51]. The primary objective of the study was to explore if patients with stage II colon cancer, who test negative for ctDNA after surgery, can be exempt from ACT without affecting their overall survival. The study included 455 patients with stage II CRC who were randomly divided into two groups: ctDNA-guided treatment and standard treatment. The ctDNA-guided group had a lower proportion of patients receiving adjuvant chemotherapy than the standard-management group (15% vs. 28%; relative risk, 1.82; 95% CI, 1.25 to 2.65), but both groups had similar 2-year RFS and met a prespecified non-inferiority endpoint (93.5% vs. 92.4%; absolute difference, 1.1% points; 95% CI, -4.1 to 6.2 [noninferiority margin, -8.5% points]). This study provides preliminary evidence of the value of MRD in guiding adjuvant therapy. ctDNA-guided treatment can effectively reduce the utilization of ACT and avoid ineffective chemotherapy in certain patients. Additionally, ongoing prospective intervention studies will further validate the value of MRD to guide treatment decisions (Table 3). In summary, ctDNA-based MRD is a biomarker for prognosis and adjuvant therapy in patients with early-stage resectable CRC.

**Table 3:** Ongoing ctdna MRD-based prospective intervention trials in CRC

Trial Name (Clinical Trial No.)	Sample Size		Study Population	Analysis Method	Study Description	Primary Objective
CIRCULATE AIO-KRK-2017 (NCT04089631)	4812		Stage II	NGS	MRD-positive patients are randomized to: Surveillance vs. ACT (capecitabine or CAPOX)	5-year DFS
CIRCULATE PRODIGE 70 (NCT04120701)	1980		Stage II	ddPCR	MRD-positive patients are randomized to: Surveillance vs. ACT (mFOLFOX6)	3-year DFS
CIRCULATE-US (NCT05174169)	1912		Stage III	Signatera	MRD-positive: mFOLFOX6 3–6 months/CAPOX 3 months/no treatment MRD-negative: mFOLFOX6 6 month/CAPOX 6 month/mFOLFOX6 + 5-FU	5-year DFS
BESPOKE (NCT04264702)	1788		Stage I-IV	Signatera	MRD-guided group: surveillance or ACT Standard of care group: traditional ACT	The impact of SIGNATERA™ on adjuvant treatment decisions
TRACC (NCT04050345) [99]	1621		High-risk Stage II and III	NGS	Standard of care group: CAPOX 3 months/Capecitabine 6 months MRD-guided group: MRD-positive, Traditional ACT; MRD-negative, chemotherapy de-escalated	3-year DFS
NRG GI-005 /COBRA (NCT04068103)	1408		Low-risk Stage IIA	LUNAR-1 (Guardant Health)	Standard of care group: Surveillance MRD-guided group: MRD-positive, traditional ACT; MRD-negative, surveillance	3-year DFS
MEDOCC-CrEATE (NL6281/NTR6455)	1320		Stage II	PGDx elio™	Standard of care group: standard follow-up according to guideline MRD-guided group: MRD-positive, 8 cycles of capecitabine plus oxaliplatin; MRD-negative, standard follow-up according to guideline	the proportion of patients receiving ACT when ctDNA is detectable after resection
VEGA-CIRCULATE (UMIN000039205)	1240		High-risk stage II and low-risk stage III	Signatera	MRD-negative patients are randomized to: CAPOX 3 months vs. Surveillance/ALTAIR study	2-year DFS
DYNAMIC-III (ACTRN-12617001566325)		1000	Stage III	Safe-SeqS	Standard of care group: traditional ACT MRD-guided group: MRD-positive, chemotherapy escalated; MRD-negative, chemotherapy de-escalated	3-year RFS
CINTS-R (NCT05601505)		465	LARC	NGS	Standard of care group: traditional nCRT MRD-guided group: MSI-H/TMB-H or POLD/POLE2 mutation willed be arranged to receive immune therapy following nCRT; The others are arranged to randomly receive TNT or nCRT	2 years of Disease-related treatment failure, DrTF
DYNAMIC (ACTRN-12615000381583)		450	Stage II	Safe-SeqS	Standard of care group: treated at the discretion of the clinicians MRD-guided group: MRD-positive, ACT; MRD-negative, surveillance	3-year RFS
DYNAMIC-RECTAL (ACTRN-12617001560381)		408	LARC	Safe-SeqS	Standard of care group: treated at the discretion of the clinicians MRD-guided group: MRD-positive, ACT; MRD-negative, surveillance/ACT	whether an adjuvant therapy strategy based on ctDNA results in addition to standard pathologic risk assessment may affect the number of patients treated with chemotherapy
IMPROVE-IT2 (NCT04084249)		254	High-risk Stage II and Stage III	ddPCR, NGS	Standard of care group: standard follow-up according to guideline MRD-guided group: MRD testing every 4 months post-op for 24 months; PET-CT every 3 months if MRD turns positive	Fraction of patients with relapse receiving curative resection or local treatment
ALTAIR-CIRCULATE (NCT04457297)		240	Resectable Stage II-IV	Signatera	MRD-positive patients after 3 months of CAPOX are randomized to: trifluridine/tipiracil vs. surveillance	3-year DFS
BNT122-01 (NCT04486378)		201	Resected Stage II (High Risk) and Stage III	Not reported	mRNA vaccine RO7198457 vs. standard treatment group MRD tested every 3 months	5-year DFS
PEGASUS (NCT04259944)		140	Resected MSS stage III and high-risk stage II (T4N0)	LUNAR-1 (Guardant Health)	MRD-positive: CAPOX 3 months MRD-negative: capecitabine 6 months but will be retested after 1 cycle, and if MRD turns positive, will be switched to CAPOX.	Number of post-surgery and post-adjuvant false negative cases after a double MRD-negative detection
NCT05062317		120	CRLM	Not reported	ctDNA (Low Risk): less intense chemotherapy ctDNA (High Risk): more intense chemotherapy	1-year RFS
NCT05635630		110	mCRC	Not reported	MRD-positive: adjuvant therapy; The ctDNA status is evaluated every 2 months. MRD-negative: Patients of MRD-negative group are monitored by ctDNA every 3 months.	2-year RFS
NCT03436563		74	Stage IV	Signatera	MRD-positive following resection of all metastases: M7824 (anti-PDL1/TGFbetaRII fusion protein) 6 doses	ORR and ctDNA clearance rate
IMPROVE-IT (NCT03748680)		64	Stage I and II	ddPCR, NGS	MRD-positive patients are randomized to: observation vs. FOLFOX/CAPOX 6 months.	3-year DFS
CLAUDE (NCT05350501)		34	Stage II/III/IV	Not reported	MRD-positive patients after curative treatment: EO2040 (peptide vaccine) + Nivolumab	Number and percentage of patients with Treatment-Emergent Adverse Events

Abbreviations: LARC, locally advanced rectal cancer; CRLM, colorectal liver metastases; mCRC, metastatic colorectal cancer; ctDNA, circulating tumor DNA; MRD, molecular residual disease; Safe-SeqS, Safe-sequencing system; NGS, Next-generation sequencing; ddPCR, droplet digital PCR; DFS, disease-free survival; RFS, recurrence-free survival; ORR, objective response rate; TMB, high tumor mutational burden; MSI, microsatellite instability; ACT, adjuvant chemotherapy; TNT, total neoadjuvant treatment; nCRT, neoadjuvant chemoradiotherapy

### ctDNA MRD for locally advanced rectal cancer

National Comprehensive Cancer Network (NCCN) guidelines recommend that locally advanced rectal cancer (LARC) patients are treated with neoadjuvant chemoradiotherapy (nCRT) followed by total mesorectal excision (TME) and postoperative adjuvant chemotherapy [52]. However, 30% of patients still experience distant recurrence after the multimodal treatment [53]. Studies indicate that for the patients achieving a clinical complete response (cCR) following neoadjuvant chemoradiotherapy (nCRT), the “Wait and Watch” (W&W) approach can reduce unnecessary surgical risks while maintaining therapeutic efficacy and significantly improve the quality of life of patients [54, 55]. Notably, approximately 16–34% of cCR patients treated with the W&W approach experience local disease recurrence [56]. Consequently, there remains a critical need for reliable biomarkers capable of identifying patients at high risk of recurrent disease and predicting which cCR patients will benefit from the W&W strategy.

Multiple studies have shown that MRD after nCRT is a biomarker of LARC prognosis (Table 4). McDuff et al. [57]reported that patients who had detectable ctDNA after surgery had poorer RFS. Tie et al. [58]demonstrated a significant reduction in RFS if ctDNA was detected after nCRT (HR, 6.6; *P* < 0.001) or after surgery (HR, 13.0; *P* < 0.001). Postoperative ctDNA-positive patients had a lower estimated 3-year RFS (33%) compared to postoperative ctDNA-negative patients (87%). Liu et al. [59] found that cases with a positive MRD detected after NAT had a significantly higher risk of recurrence. Similarly, Dizdarevic et al. [60]performed a systematic review of nine studies on ctDNA in LARC and found that ctDNA positivity at baseline, before or after surgery was negatively associated with survival. Patients with LARC exhibiting detectable ctDNA at multiple time points have an increased risk of recurrence.

**Table 4:** CtDNA MRD studies in LARC

Reference	Sample Size	Analysis Method	Neoadjuvant Chemotherapy	Adjuvant Chemotherapy	% of Patients ctDNA Positive	Main Findings
Ji et al., 2021 [88]	46	NGS	100%	100%	Not reported	• No significant correlation between cfDNA levels and tumor recurrence or OS at baseline, post nCRT, or post-operation. • Patients with decreasing TMB tend to have a longer median RFS after CRT (*P* = 0.022). • bTMB level after surgery was negatively correlated with RFS (*P* = 0.026).
Khakoo et al., 2020 [90]	47	ddPCR	100%	91.3%	Baseline: 71% Post-CRT: 21% Post-op: 13% Surveillance: not reported	All 3 patients with detectable MRD post-surgery relapsed compared with none of the 20 patients with undetectable MRD (*P* = 0.001).
Liu et al., 2022 [59]	60	PCR	100%	Not reported	Post-CRT: 23.3% Surveillance: not reported	Positive MRD post-CRT personalized assay was significantly associated with an increased risk of recurrence (HR, 27.38; log-rank *P* < 0.0001).
McDuff et al., 2021 [57]	29	ddPCR	100%	Not reported	Baseline:31% Post-op: 21% Post-Treatment: not reported Surveillance: not reported	Patients with detectable postoperative MRD experienced poorer RFS than those with undetectable MRD (HR, 11.56;*P* = 0.007), PPV = 100%, NPV = 87%.
Murahashi et al., 2020 [87]	85	Amplicon-based deep sequencing	100%	Not reported	Baseline: 57.6% Post-nCRT: 22.3%	• Change in ctDNA was an independent predictor of complete response to preoperative therapy (*P* = 0.0276). • Postoperative MRD and CEA were independent prognostic markers for the risk of postoperative recurrence (MRD, *P* = 0.0127, CEA, *P* = 0.0105)
Ravenda et al., 2018 [63]	28	NGS	100%	Not reported	Not reported	Patients with pCR (25%) had lower pre-treatment ctDNA values (median of 0.0) compared to patients with residual tumors (median value of 0.11, *P* = 0.18) and an AUC of 0.67 (95% CI: 0.47–0.87). Similar results were observed for post-treatment ctDNA values.
Schou et al., 2018 [86]	123	Direct fluorescence	100%	42%	Not reported	No difference was observed post-treatment circulating free DNA (cfDNA) levels between patients with pCR and poor responders.
Tie et al., 2019 [58]	159	Safe-SeqS	100%	64.2%	Post-op: 11.9% Post-Treatment: 8.3% Surveillance: not reported	• Detectable MRD was associated with poor RFS after chemoradiotherapy (HR, 6.6; *P* < 0.001) or after surgery (HR, 13.0; *P* < 0.001). • The 3-year RFS was 33% for detectable MRD patients and 87% for undetectable MRD patients after surgery.
Vidal et al., 2021 [99]	72	NGS	100%	Not reported	Baseline: 83% Pre-op:15%	• ctDNA status was not associated with pathologic response. • Detectable MRD pre-surgery was significantly associated with systemic recurrence, shorter DFS (HR, 4; *P* = 0.033) and shorter OS (HR, 23; *P* < 0.0001).
Wang et al., 2021 [64]	119	NGS	100%	Not reported	Not reported	• Detection of potential CRC driver genes in ctDNA after nCRT indicated a significantly worse RFS (HR, 9.29; 95% CI, 3.74 to 23.10; *P* < 0.001). • Patients with detectable driver mutations and positive high-risk feature after surgery had the highest recurrence risk (HR, 90.29; 95% CI, 17.01 to 479.26; *P* < 0.001).
Wang et al., 2023 [100]	119	NGS	100%	Not reported	Not reported	Based on the 5’-end motif profile plus mrTRG achieved the highest cross-validation AUC (0.92, 95% CI, 0.91–0.93) and the combination of a 5’-end motif profile with mrTRG has the potential to predict the response to nCRT.
Yang et al., 2019 [101]	119	NGS	100%	Not reported	Not reported	Detection MRD of pre-treatment mutations after completion of nCRT was significantly associated with worse DFS (*P* < 0.05).
Zhou et al., 2021 [62]	106	HiSeq 3000 Sequencing System (Illumina)	99%	80.6%	Baseline: 75% Pre-op:10.5% Post-op: 6.7% Post-Treatment: not reported Surveillance: not reported	None of the 29 patients with pathologic complete response (ypCR) had preoperative ctDNA detected and also that *POLD1* mutations and higher tumor mutation burden could also help predict good response.
Zitte et al., 2008 [61]	26	real-time PCR	69%	Not reported	Not reported	Before and after chemoradiation, responders and non-responders had comparable median plasma cfDNA values (7.3 ng/ml vs. 3.6 ng/ml, *P* = 0.693; 2.6 ng/ml vs. 1.0 ng/ml, *P* = 0.340).

Abbreviations: LARC, locally advanced rectal cancer; ctDNA, circulating tumor DNA; cfDNA, cell-free DNA; MRD, molecular residual disease; Safe-SeqS, Safe-sequencing system; NGS, Next-generation sequencing; ddPCR, droplet digital PCR; WGS, Whole genome sequencing; HR, hazard ratio; CI, confidence interval; DFS, disease-free survival; RFS, recurrence-free survival; OS, overall survival; Pre-op, pre-operative; Post-op, post-operative; TMB, high tumor mutational burden; nCRT, neoadjuvant chemoradiotherapy; pCR, pathological complete response; CEA, carcinoembryonic antigen

In the past studies, the conclusions of ctDNA in predicting pathological complete response (pCR) were inconsistent (Table 4). Zitt et al. [61]for the first time showed an association between pathological response and cfDNA concentrations in pre- and post-nCRT. Another study showed that patients who achieved pathological complete response (ypCR) had no detectable preoperative ctDNA [62]. In one study with 28 patients’ initial outcomes, patients who achieved pCR (25%) had the lowest median pre-treatment ctDNA value of 0.0, whereas patients with residual tumor had a median value of 0.11 (*P* = 0.18), with an AUC of 0.67 (95% CI: 0.47–0.87). Post-treatment ctDNA levels revealed similar results [63]. A study involving 159 patients who underwent nCRT and TME revealed no significant correlation between pre- and post-nCRT ctDNA levels and pCR. In addition, another study has demonstrated that the integration of ctDNA levels, ctDNA clearance during NCRT, and MRI tumor regression grade (mrTRG) can be superior in predicting pCR compared to ctDNA alone [64]. The current findings are primarily derived from limited-scale studies. In order to enhance the prediction of pCR status and facilitate patient selection for the W&W strategy in LARC, it is imperative to conduct more extensive studies on ctDNA and explore predictive models that integrate ctDNA with other clinical variables.

ctDNA is a promising biomarker with the potential to guide adjuvant chemotherapy for locally advanced rectal cancer. The DYNAMIC-Rectal study [65] found that ctDNA-guided therapy reduces the use of chemotherapy in patients with LARC. Patients who underwent nCRT and TME for locally advanced rectal cancer were randomly assigned to either ctDNA-guided or standard-management groups at a ratio of 2:1. The proportion of patients receiving adjuvant chemotherapy based on ctDNA-guided (*n* = 155) and standard-management (*n* = 75) was 46% and 77%, respectively (*p* < 0.001). At a median follow-up of 36 months, 3-year RFS was 74% for ctDNA-guided and 82% for standard-management, with no significant difference between the two groups.

In conclusion, ctDNA MRD is a valuable tool for predicting response and prognosis in LARC patients who receive nCRT and surgery. It has the potential to assist patients in selecting more appropriate treatments.

### ctDNA monitoring for metastatic colorectal cancer

Approximately 20% of patients with CRC are diagnosed with metastatic colorectal cancer (mCRC) at their initial diagnosis [66]. Although treatment algorithms have improved, the 5-year survival rate for mCRC remains below 20% [67, 68]. More than 50% of patients with mCRC experience relapse after surgery due to the presence of MRD [69, 70]. The level of ctDNA after surgery can indicate the presence of MRD, identifies patients with a high risk of recurrence, and may be more sensitive than CT imaging [71]. Several studies have shown that postoperative ctDNA-positive is associated with poorer prognosis in resectable mCRC (Table 5). A retrospective analysis performed ctDNA-based MRD detection of plasma samples from 69 mCRC patients who underwent surgical resection and the results indicated that ctDNA-positive was related to lower DFS (HR, 4.97; 95% CI, 2.67–9.24; *P* < 0.0001) and OS (HR, 27.05; 95% CI, 3.60-203.46; *P* < 0.0001) [72]. Similarly, a separate study [73] revealed that mCRC patients who were ctDNA-positive after surgery or adjuvant chemotherapy experienced reduced RFS than those without detectable ctDNA (HR, 4.5; *P* < 0.0001 vs. HR, 8.4; *P* < 0.0001). Another study [74]showed that patients who were MRD-negative at two time points (postsurgical plus last follow-up time point) had an OS of 100%, even without systemic treatment. Patients who test positive for MRD have a lower overall survival. The above study confirms that ctDNA after surgery or adjuvant chemotherapy is a strong prognostic biomarker for mCRC.

**Table 5:** CtDNA MRD studies in advanced CRC

Reference	Sample Size	Cancer Stage		Analysis Method	Neoadjuvant Chemotherapy			Adjuvant Chemotherapy	% of Patients ctDNA Positive	Main Findings
Benešová et al., 2019 [102]	47	IV		PCR	Not reported			Not reported	Post-op: 7% Post-Treatment: Not reported Surveillance: Not reported	Patients with positive MRD after R0 resection (2/28) were diagnosed with a recurrence of the disease after 6 months.
Bratman et al., 2020 [80]	94	IV		NGS	Not reported			Not reported	Not reported	All 12 patients experienced prolonged objective responses and 100% OS with a median of 25.4 (range 10.8–29.5) months of follow-up beyond first clearance.
Lee et al., 2021 [81]	67	IV		ICP v2	53.7%			Not reported	Pre-op: 23.9% Post-op: 16.4%	ctDNA detection rate tended to be low in cases of good response to chemotherapy.
Levy et al., 2012 [103]	7	III/IV	fluorescently labeled PCR			25%		75%	Pre-op: 100% Post-op < 1week: 28.6% Post-op < 6months: 28.6% Post-op < 6-12months: 28.6% Post-op < 12-18months: 57.1% Post-op < 18-24months: 14.3%	The tumor cfDNA status was found to be always closely correlated with the actual clinical status of the patient.
Lonardi et al., 2022 [72]	69	IV (resected)		FoundationOne^®^Tracker			42.0%	Not reported	Post-op: 45.0% Post-Treatment: 35.3% Surveillance: Not reported	MRD-positive post curative-intent surgery was significantly associated with lower DFS (HR, 4.97; 95% CI, 2.67 − 9.24; *P* < 0.0001) and OS (HR, 27.05; 95% CI, 3.60 − 203.46; *P* < 0.0001).
Loupakis et al., 2021 [74]	112	IV (liver resection)		Signatera	Not reported			39.2%	Post-op: 54.4% Post-Treatment: Not reported Surveillance: Not reported	MRD-positive status was associated with an inferior OS: HR, 16.0; 95% CI, 3.9 to 68.0; *P* < 0.001.
Murray et al., 2018 [104]	172	III-IV		Real-time qPCR assay (methylated *BCAT1* and *IKZF1*)	Not reported			Not reported	Post-op: 16% Post-Treatment: Not reported Surveillance: Not reported	MRD-positive was independently associated with an increased risk of recurrence (HR, 3.8; *P* = 0.004).
Overman et al., 2017 [105]	54	IV (resectable CRLM)		a novel 30 kb ctDNA digital sequencing panel (Guardant Health)	Not reported			Not reported	Post-op: 44% Post-Treatment: Not reported Surveillance: Not reported	• Detectable MRD post-op was correlated with RFS (*P* = 0.002, HR, 3.1; 95% CI 1.7–9.1) with 2-year RFS of 0% vs. 47%. • Recurrence was detected in ctDNA at a median of 5.1 months before radiographic recurrence.
Pellini et al., 2021 [10]	24	oligometastatic CRC		NGS	71%			Not reported	Not reported	mCRC patients with MRD after nCRT may benefit from personalized treatment based on ctDNA genomic profiling.
Øgaard et al., 2022 [73]	96	IV (resectable CRLM)		ddPCR	9.3%			43.7%	Pre-op:85.4% Post-op:40.6% Post-Treatment: 16.7% Surveillance: 60.4%	Patients with detectable MRD postoperatively had a significantly lower RFS than patients with undetectable MRD (HR, 4.5; *P* < 0.0001).
Tie et al., 2015 [106]	53	IV (treatment-naive)		Safe-SeqS	0%			100%	Not reported	Major reductions (≥ 10-fold) versus lesser reductions in ctDNA precycle 2 were associated with a trend for increased PFS (median 14.7 versus 8.1 months; HR, 1.87; *P* = 0.266).
Tie et al., 2021 [107]	54	IV (resectable CRLM)		Safe-SeqS	43%			78%	Baseline: 85% Post-op: 24% Post-Treatment: 25% Surveillance: 87.5%	Patients with detectable MRD postoperative had a lower RFS (HR 6.3; 95% CI 2.58 to 15.2; *P* < 0.001) and OS (HR, 4.2; 95% CI 1.5 to 11.8; *P* < 0.001).
Wang et al., 2021 [108]	91	IV (resectable CRLM)		NGS	92.3%			91.2%	Baseline: 88.7% Post-op: 41.0% Post-Treatment: 44.9% Surveillance: 79.4%	A significant difference in overall recurrence rate was observed in patients with detectable vs. undetectable MRD after resection of CRLM (79.4% vs. 41.7%).
Zou et al., 2020 [109]	28	IV (resectable CRLM)		ddPCR	Not reported			Not reported	Twenty-eight patients who underwent potentially curative surgery and had no evidence of residual disease were monitored for up to 2 years for ctDNA.	Clinical recurrence was observed in 6/28 (21%) patients. Four of the 6 patients had a significant increase in ctDNA at or before recurrence.

Abbreviations: CRC, colorectal cancer; CRLM, colorectal liver metastases; ctDNA, circulating tumor DNA; cfDNA, cell-free DNA; MRD, molecular residual disease; Safe-SeqS, Safe-sequencing system; NGS, Next-generation sequencing; ddPCR, droplet digital PCR; HR, hazard ratio; CI, confidence interval; RFS, recurrence-free survival; PFS, progression-free survival; OS, overall survival; Pre-op, pre-operative; Post-op, post-operative; nCRT, neoadjuvant chemoradiotherapy

MRD was evaluated with a methylation-based ctDNA assay in 118 resectable colorectal oligometastases patients [75]. Patients with a poor genomic profile were determined to possess at least one of the BRAF, RAS, PIK3CA, or SMAD4 mutations. It was found that patients with both a poor genomic profile and positive ctDNA after surgery had a significantly shorter RFS and could benefit from adjuvant chemotherapy. However, no significant associations with adjuvant chemotherapy were observed for the other patient groups.

For the unresectable mCRC, ctDNA MRD is currently used to monitor disease progression and predict therapeutic efficacy (Table 5). A prospective study enrolled 171 treatment-naive mCRC patients using serial ctDNA testing to investigate the correlation between ctDNA and the outcome of systemic therapy [76]. The study showed that patients with plasma RAS/BRAF clearance had better outcomes compared to those who had the RAS/BRAF mutation. Iris et al. [77] used cfDNA and WBC combined analysis metastatic colorectal cancer treatment response. Longitudinal analysis of ctDNA is more predictive of OS compared to conventional standard imaging assessments. The ctDNA testing enabled the screening of 42% of patients for primary or acquired resistance to panizumab, facilitating early identification of drug-resistant stages and timely adjustment of treatment regimens. Thomsen et al. [78] analyzed meth-ctDNA before start of treatment and before the second cycle in 123 patients with metastatic CRC. The study showed patients with low meth-ctDNA after the first treatment cycle had a median survival of 25.4 months compared to 13.5 months in the group with meth-ctDNA. The results suggest that ctDNA response has potential as a surrogate marker of OS. However, further validation is needed for its clinical application.

Another potential clinical application of ctDNA is the identification of non-responders to immunotherapy. The KEYNOTE-177 study showed that immunotherapy prolongs PFS in patients with high microsatellite instability (MSI-H) CRC. However, approximately 30% of patients do not respond to immunotherapy. If ctDNA is used early in treatment to identify patients who do not respond to immunotherapy, it may enable receive chemotherapy earlier and potentially improve prognosis [79]. A prospective Phase II clinical trial evaluated ctDNA levels in five distinct cohorts of advanced solid tumor patients treated with pembrolizumab [80]. The researchers analyzed the levels of ctDNA in patients at the beginning of cycle 3 of treatment with pembrolizumab. Out of the 73 patients, 33 exhibited a decrease in ctDNA levels compared to baseline, and among them, 14 achieved an objective response. Among the remaining 40 patients whose ctDNA levels increased from baseline, only one had an objective response (OR: 28.74). Another study on PD-1 inhibitors found that patients who experienced an increase in ctDNA levels at four weeks of treatment showed tumor progression at two months, while those with decreased ctDNA levels maintained stable disease. These studies demonstrate that dynamic monitoring of ctDNA MRD can determine the efficacy of immunotherapy and predict the potential “cure” population for late-stage immunotherapy. In conclusion, ctDNA MRD is a promising biomarker for mCRC that can provide real-time information on tumor dynamics and molecular characteristics. It has shown clinical utility in predicting prognosis and treatment response in patients with mCRC.

### Challenges and prospects

Despite the promising results of ctDNA detection in CRC, there are still several challenges and limitations that need to be addressed. First, the sensitivity of ctDNA testing depends on various factors. For patients with early-stage CRC who responded satisfactorily to chemotherapy after tumor removal, the majority of MRD is eliminated or present at undetectable levels [81]. In patients with mCRC, ctDNA testing in patients with brain metastases or peritoneal carcinomatosis is limited due to the plasma-peritoneal barrier or blood-brain barrier. Tie et al. [82] indicated that postoperative false-negative MRD results were more frequent in patients with peritoneal omental relapse compared to those with distant relapse. There are inconsistent mutation statuses between tissues and plasma. Vidal et al. [83] analyzed RAS mutations in both tissue and plasma samples from patients with metastatic colorectal cancer, revealing that the site of metastasis can lead to inconsistencies in mutations detected in blood and tissues. There are also studies that show that ctDNA was undetectable in preoperative plasma samples in some CRC patients, suggesting that not all tumors shed ctDNA. There are many reasons for low ctDNA shedding, such as the tumor burden, tumor size, location, and cell turnover [84]. All of these factors limit the sensitivity of ctDNA testing. As mentioned earlier, serial ctDNA testing can improve detection rates for ctDNA MRD. Recent research shows that integrating epigenomic signatures enhances sensitivity by 25-36% when combined with genomic alterations alone [33]. In the future, we can utilize serial ctDNA testing or multiomics to improve detection sensitivity. Second, there is a lack of reported best practices for the duration of MRD testing in patients with CRC. A study of non-small cell lung cancer (NSCLC) found the peak risk of developing detectable MRD was approximately at 18 months after surgery, suggesting longitudinal undetectable MRD may define the patients who were cured [85]. More prospective studies are needed to establish standardized protocols and guidelines for MRD testing in CRC patients. Third, the clinical significance and utility of ctDNA MRD detection have not been fully validated in prospective trials or real-world settings, and it remains unclear whether MRD can be used as a prognostic surrogate endpoint [57, 58, 86–88]. Most studies of MRD in different solid tumors have focused on prognosis and are observational, with little data reported on MRD-based interventional studies. It is hoped that with the continuous development and maturation of MRD technology, more prospective and interventional studies in the future to test the reliability of MRD technology and to further demonstrate the value for clinical decision-making. Fourth, the cost-effectiveness and accessibility of ctDNA MRD testing are not well evaluated and may pose barriers to their widespread adoption in clinical practice. Few studies have evaluated the cost-effectiveness of MRD testing in CRC. One study from Australia estimated that MRD testing would cost A$ − 4055 (− 16,853 to 8472) and A$ − 2284 (− 14,685 to 10,116), and would be cost-effective at a threshold of £20,000 per quality-adjusted life-year (QALY) gained [89]. MRD testing may be a cost-effective strategy for CRC management, but more data are needed to confirm these results and account for uncertainty. As for accessibility, it will involve the availability and affordability of healthcare services for different populations. Several factors may affect the accessibility of MRD testing in CRC, such as the availability of laboratory facilities, the turnaround time of test results, the reimbursement policies of health insurance providers, and the awareness and acceptance of patients and physicians. MRD testing requires high-throughput sequencing platforms and bioinformatics pipelines that may not be widely available or standardized across different settings. Furthermore, MRD testing may not be covered by health insurance plans or may require high out-of-pocket payments from patients. Additionally, MRD testing may face challenges in terms of patient and physician education, consent, and communication. Therefore, several barriers may limit the accessibility of MRD testing in CRC.

## Conclusion

In conclusion, ctDNA based MRD detection is emerging as a novel biomarker for CRC diagnosis and management. MRD analysis not only provides more accurate stratification of risk of recurrence but also allows adjustment of the intensity and duration of adjuvant therapy based on the test results. Moreover, ctDNA MRD monitoring may predict the effectiveness of adjuvant therapy and improve the efficiency of adjuvant trials. Overall, ctDNA based MRD has the potential to improve risk stratification, treatment selection, response evaluation, relapse prediction, and molecular surveillance in patients with CRC.

## Data Availability

No datasets were generated or analysed during the current study.
